# ASSOCIATION BETWEEN SARCOPENIA AND NEUROPSYCHIATRIC SYMPTOMS IN OLDER ADULTS WITH ALZHEIMER’S DISEASE

**DOI:** 10.1093/geroni/igae098.3797

**Published:** 2024-12-31

**Authors:** Derong Zeng, Nan Zhang, Bingbing Qi, Teruaki Kawasaki, Ichiro Akiguchi, Ayae Kinoshita

**Affiliations:** Kyoto University, Kyoto City, Kyoto, Japan; The University of Manchester, Manchester, England, United Kingdom; Villanova University, Villanova University, Pennsylvania, United States; Kyoto Clinical and Translational Research Center for Neurocognitive Disorders, Kyoto Clinical and Translational Research Center for Neurocognitive Disorders, Kyoto, Japan; Kyoto Clinical and Translational Research Center for Neurocognitive Disorders, Kyoto Clinical and Translational Research Center for Neurocognitive Disorders, Kyoto, Japan; Kyoto University, Kyoto University, Kyoto, Japan

## Abstract

Sarcopenia is a vulnerability state that increases the risk of many adverse health outcomes, but little is known about the effects of sarcopenia on neuropsychiatric health. To explore the association between sarcopenia and neuropsychiatric symptoms in older adults with Alzheimer’s Disease, data from 110 participants were assessed using the Asian Working Group for Sarcopenia criteria. The participants had a mean age of 83.15 years (SD = 5.49), a mean MMSE score of 20.69 (SD = 5.03). The study focused on the relationship between sarcopenia and 12 neuropsychiatric inventory items, including delusions, hallucinations, aggression, depression, anxiety, elation, indifference, disinhibition, lability, aberrant motor behaviors, nighttime disturbances, and eating disturbances. The relationships between sarcopenia and these neuropsychiatric symptoms were explored using logistic regression models. Logistic regression analyses revealed significant associations between sarcopenia and both aberrant motor behaviors (Estimate=-1.5612, p=0.0141) and nighttime disturbances (Estimate= -1.2096, p=0.0194). Further analysis incorporating age, sex, MMSE, and grip strength showed that while the association between sarcopenia and eating disturbances was not significant (Estimate=1.51272, p=0.1248), age emerged as a significant predictor (Estimate = 0.18272, p = 0.00869), suggesting that age may be a more critical factor in the development of eating disturbances in this population. These findings suggest that sarcopenia may play a role in the development or exacerbation of specific neuropsychiatric symptoms in AD patients. Further research is needed to understand the underlying mechanisms and to develop targeted interventions to improve the quality of life for this population.

